# A cross-kingdom history

**DOI:** 10.7554/eLife.07527

**Published:** 2015-04-15

**Authors:** Alisdair R Fernie, Takayuki Tohge

**Affiliations:** Max Planck Institute of Molecular Plant Physiology, Potsdam-Golm, Germanyfernie@mpimp-golm.mpg.de; Max Planck Institute of Molecular Plant Physiology, Potsdam-Golm, Germanytohge@mpimp-golm.mpg.de

**Keywords:** vitamin C, ascorbate, *Porphyra*, L-gulonolactone oxidase, *Galdieria*, other

## Abstract

The enzyme that catalyses the last step in the synthesis of ascorbate has been repeatedly lost and replaced during the evolution of the different kingdoms of eukaryotes.

**Related research article** Wheeler G, Ishikawa T, Pornsaksit V, Smirnoff N. 2015. Evolution of alternative biosynthetic pathways for vitamin C following plastid acquisition in photosynthetic eukaryotes. *eLife* **4**:e06369. doi: 10.7554/eLife.06369

**Image** Ascorbate is a powerful antioxidant that is synthesised via different metabolic pathways

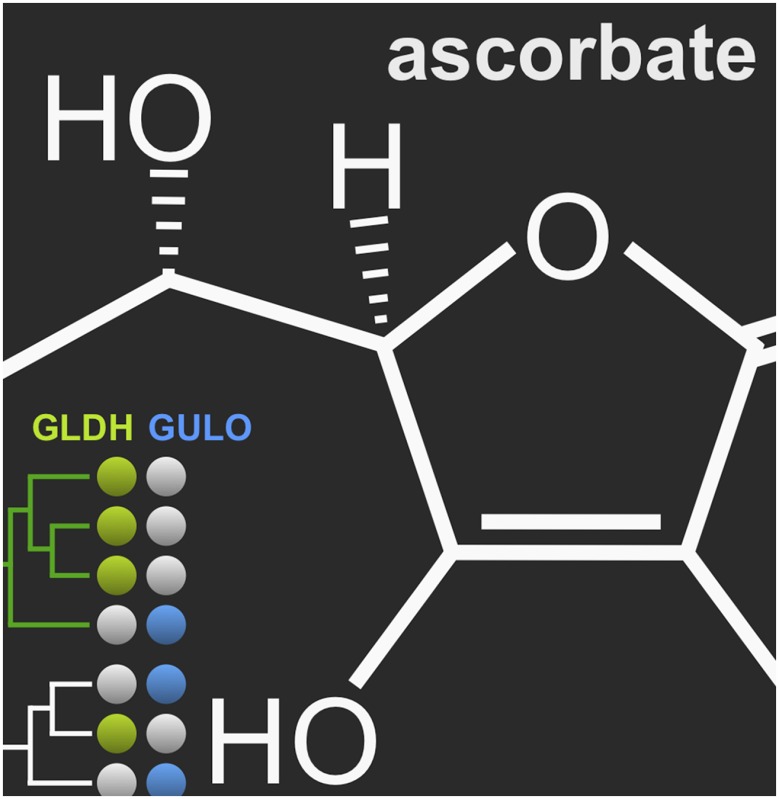

Between two and three billion years ago, living things evolved the ability to use oxygen to increase the amount of energy that could be released from carbon-rich molecules. However, these reactions produce harmful molecules called ‘reactive oxygen species’ as by-products, so antioxidant systems had to be developed in parallel to detoxify them (Noctor and Foyer, 1998; Gest et al., 2013).

A compound called ascorbate is an excellent antioxidant that is made by almost all living organisms. However, certain animals—including guinea pigs (Nishikimi et al., 1992), most bats, some fish and bird species, as well as humans and many other primates—have all have lost the ability to synthesise this compound for themselves (Mandl et al., 2009). Instead, these animals must obtain ascorbate (which is also known as ‘vitamin C’) from their diets. Moreover, all of these animals are unable to make ascorbate for the same reason: they have all lost a gene that encodes an enzyme called gulonolactone oxidase (or GULO). This enzyme catalyses the final step in the metabolic pathway that produces ascorbate in other animals.

Three major routes of ascorbate biosynthesis have been described across the kingdoms of life. The animal kingdom uses one of these pathways, and photosynthetic eukaryotes—such as plants, algae and some single-celled organisms—employ the other two. All three pathways proceed via different intermediate molecules, but the two from photosynthetic eukaryotes both use an enzyme called galactolactone dehydrogenase (or GLDH), instead of GULO, to catalyse the final step (Wheeler et al., 1998).

Now, in *eLife*, Glen Wheeler (Marine Biological Association), Takahiro Ishikawa (Shimane University), and Varissa Pornsaksit and Nicholas Smirnoff (both at the University of Exeter) have analysed the distribution of these two enzymes amongst the genomes of many different eukaryotes to determine the origins of ascorbate biosynthesis (Wheeler et al., 2015). They found that each genome either contained a gene for GULO or a gene for GLDH, but not both (Figure 1A). They also revealed that the ancestor of all animals and fungi (and their close relatives) likely used GULO, and that this ancestral trait has been lost in many later groups, including all insects.

**Figure 1.**
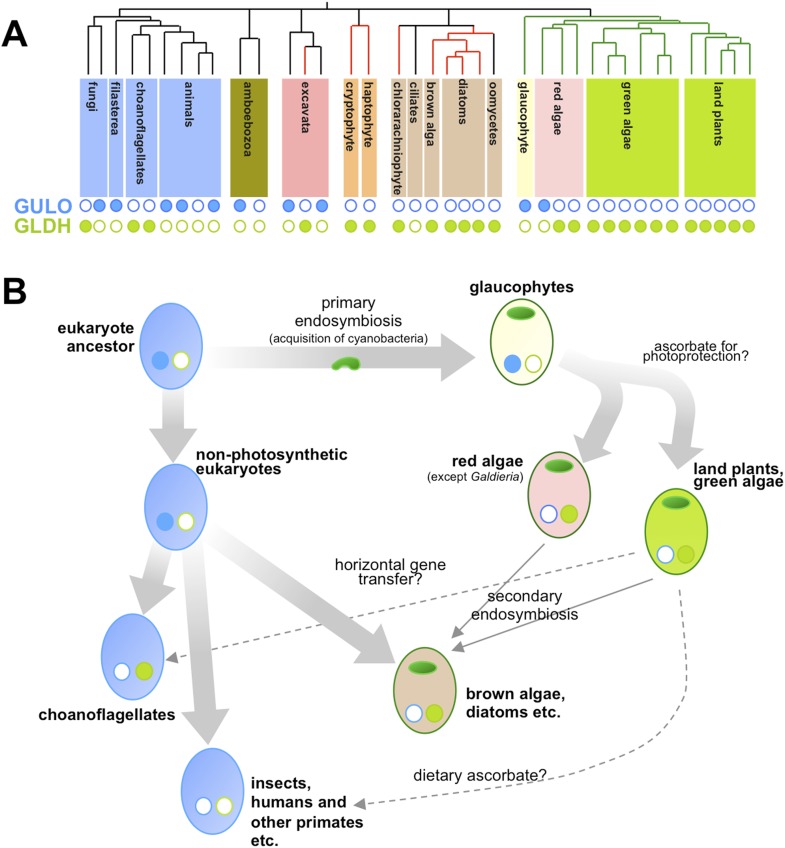
Taxonomic distribution and evolutionary history of ascorbate biosynthesis. (**A**) The phylogenetic tree depicts the currently accepted evolutionary relationships between the major lineages of eukaryotes. Black vertical lines indicate non-photosynthetic groups, whilst green lines indicate organisms that became photosynthetic after acquiring an ancestral cyanobacterium (via a so-called ‘primary endosymbiosis event’). Red lines indicate organisms that became photosynthetic after acquiring a red alga or green alga (via so-called ‘secondary endosymbiosis events’). Groups with a copy of the gene for an enzyme called GULO in their genome are marked with a closed blue circle, whilst those with a copy of the gene for an enzyme called GLDH are marked with a closed green circle. Empty circles indicate that the gene is absent from the genome. (**B**) The schematic model suggests how the primary routes of ascorbate biosynthesis arose in the various eukaryotic lineages and offers potential explanations based on Wheeler et al.'s findings. The presence and absence of GULO and GLDH are indicated as above, and chloroplasts (which are originally derived from the cyanobacterial ancestor) are indicated via green ovals. The wide arrows indicate the evolutionary relationships between groups. The thin arrows indicate the acquisition of a red or green alga by a non-photosynthetic eukaryote and dashed arrows represent events that may explain the gain or loss of genes involved in ascorbate biosynthesis.

Ascorbate biosynthesis via GULO also occurs in several other groups of eukaryotes, including a primitive, freshwater alga called *Cyanophora paradoxa* and two species in a single genus of red algae (called *Galdieria*). However, the gene for GULO is absent in the genomes of the vast majority of other algae and the land plants. Instead, Wheeler et al. revealed that almost all photosynthetic eukaryotes use the GLDH enzyme. Gene expression datasets confirmed that this enzyme is also found in non-photosynthetic organisms that are descended from photosynthetic ancestors—namely the water moulds (or oomycetes). Moreover, while several non-photosynthetic organisms (including the ciliates and an amboebozoan) lacked both genes, this was not the case for any photosynthetic eukaryote.

Wheeler et al. also provide strong evidence that the genes for GULO and GLDH have each only arisen once—and other evidence suggests that it is possible that the two enzymes are originally descended from a single gene. This implies two possible scenarios. Firstly, GULO and GLDH could result from a single gene that was duplicated in the last common ancestor of all eukaryotes, and different lineages have subsequently lost either one gene or the other. Secondly, the two genes might have evolved in different lineages and passed between species via ‘horizontal gene transfer’. However, whilst compelling arguments are put forward for both cases, it is not currently possible to exclude either of these explanations or, indeed, a combination of both in different lineages.

Nevertheless, their comprehensive analyses allowed Wheeler et al. to speculate on the selective pressures that might have driven the evolutionary path of ascorbate biosynthesis in eukaryotes (Figure 1B). Reactive oxygen species are produced when energy from sunlight is harvested during photosynthesis. This means that photosynthetic organisms experience ‘photo-oxidative stress’ and need more efficient antioxidant systems. As such, Wheeler et al. put forward an attractive hypothesis that GULO was lost in eukaryotes that became photosynthetic because this enzyme also produces some reactive oxygen species (namely hydrogen peroxide). Photosynthetic eukaryotes switched to use GLDH instead because this enzyme can synthesise ascorbate without producing reactive oxygen species as by-products. The fact that the *Galdieria* red algae have retained GULO can also be explained since it grows on or inside porous rocks in hot sulphur springs where photo-oxidative stress is believed to be minimal.

Studying other enzymes involved in the ascorbate antioxidant system revealed that *C. paradoxa* is the only photosynthetic organism to lack these enzymes. However, photosynthetic bacteria (i.e., cyanobacteria) don't appear to use ascorbate to protect against photo-oxidative stress. This suggests that the ‘photoprotective’ role of ascorbate may have emerged in photosynthetic eukaryotes after *C. paradoxa* (and other glaucophytes) diverged from the lineages that went on to be come the red algae, the green algae and the land plants.

Finally, the evolution of a photoprotective role of ascorbate would have increased the amount of this compound found in the leaves of land plants. As such the loss of GULO in several animal lineages may well reflect the increased availability of ascorbate in their largely plant-based diets.

Wheeler et al. thus provide major insight into the evolution of ascorbate metabolism in eukaryotes. In addition, when taken together with recent studies concerning the evolution of other antioxidants (Tohge et al., 2013) and plant hormones (Ju et al., 2015), the enormous potential resulting from the torrent of information provided by modern genome sequencing technologies becomes apparent (Tautz et al., 2010). In the coming years, one can anticipate that the widespread adoption of similar approaches will enable a far greater understanding of the evolution of both fundamental and specialised metabolic pathways in all kingdoms of life.
